# Erythema induratum of Bazin associated with Addison’s disease: first description

**DOI:** 10.1590/S1516-31802012000600008

**Published:** 2013-01-18

**Authors:** Rodrigo Antonio Brandão, Jozélio Freire de Carvalho

**Affiliations:** I MD. Attending Physician, Hospital das Clínicas (HC), Faculdade de Medicina da Universidade de São Paulo (FMUSP), São Paulo, Brazil.; II MD, PhD. Collaborating Professor, Rheumatology Division, Hospital das Clínicas (HC), Faculdade de Medicina da Universidade de São Paulo (FMUSP), São Paulo, Brazil.

**Keywords:** Addison disease, Adrenal insufficiency, Tuberculosis, Erythema induratum, Tuberculosis, cutaneous

## Abstract

**CONTEXT:**

Erythema induratum of Bazin (EIB) is considered to be a tuberculid reaction and consists of recurrent painful nodules. The differential diagnosis includes diseases like nodular vasculitis, perniosis, polyarteritis nodosa and erythema nodosum.

**CASE REPORT:**

We report the case of a woman with EIB who developed Addison’s disease during treatment with anti-tuberculosis drugs with good response to glucocorticoid replacement. The diagnosis was obtained through the clinical picture, positive tuberculin test and positive BCG (bacillus Calmette-Guérin) test on the histological sample. Anti-tuberculosis drugs and glucocorticoid replacement led to disappearance of the signs and symptoms.

**CONCLUSIONS:**

This is the first description of an association between EIB and Addison’s disease. It should be borne in mind that tuberculosis is an important etiological factor for Addison’s disease.

## RESUMO

**CONTEXTO:**

O eritema indurado de Bazin (EIB) é considerado uma reação tuberculoide e consiste de nódulos dolorosos recorrentes. O diagnóstico diferencial inclui doenças como vasculite nodular, perniose, poliarterite nodosa e eritema nodoso.

**RELATO DE CASO:**

Nós reportamos o caso de uma mulher com EIB que desenvolveu doença de Addison durante tratamento com drogas tuberculostáticas e com boa resposta com reposição de glicocorticoide. O diagnóstico foi realizado pela apresentação clínica, teste tuberculínico positivo e positividade para BCG (bacilo Calmette-Guérin) no espécime histológico. A reposição de glicocorticoides e drogas tuberculostáticas levou ao desaparecimento dos sinais e sintomas.

**CONCLUSÕES:**

Este é o primeiro caso reportando a associação entre EIB e doença de Addison. Tuberculose é um importante agente etiológico da doença de Addison.

## INTRODUCTION

Erythema induratum of Bazin (EIB), also known as Bazin disease, tuberculosum, tuberculosis cutis indurativa and nodose tuberculid, is a chronic, nodular eruption that usually occurs on the lower legs of young women. It has been correlated with manifestations of tuberculin hypersensitivity consisting of a type of tuberculid that occurs on the legs, whereas nodular vasculitis represents the non-tuberculous counterpart. The number of reports of EIB has been decreasing in most developed countries in accordance with the decreasing incidence of tuberculosis. However, no previous case of EIB associated with adrenal insufficiency has been described. In this paper, we report the first case of a patient with EIB who developed adrenal insufficiency.

## CASE REPORT

The patient was a 45-year-old Caucasian female who had a history of systemic arterial hypertension and diabetes mellitus that started 10 years earlier and was treated with neutral protamine Hagedorn (NPH) insulin and enalapril. She reported having painful and erythematous nodules on the backs of her legs, together with fatigue.

Physical examination demonstrated that she was afebrile (temperature 35.7 °C) and presented subcutaneous nodules of two centimeters and three centimeters in diameter on the posterior and medial faces of both legs, with marked erythematous skin above the lesions. A biopsy on one of the lesions demonstrated caseous necrotic granulomatous panniculitis compatible with EIB. BCG (bacillus Calmette-Guérin) immunohistochemical tests were positive for this antigen. A tuberculin test showed nodulation of 15 mm in her left arm. Serological tests for HIV, syphilis and hepatitis B were negative. Investigations on tuberculosis sites were completely negative and included x-ray and computed tomography of the chest and abdomen, urine culturing (three different samples), Ziehl-Neelsen staining on sputum (three different samples), and gastric fluid investigations. She was treated with rifampicin, isoniazid and pyrazinamide.

Ten days after anti-tuberculosis therapy was introduced, she experienced asthenia, depressive mood and dizziness when standing up. She did not have any orthostatic hypotension. Sertraline treatment was started but she did not show any response until 300 mg/day was reached.

She was hospitalized on three occasions due to heart failure and hyponatremia (sodium: 125 mg/dl). During the last hospitalization, insulin administration was completely stopped since she was showing good glucose control. Six months later, she started to present facial hyperpigmentation, and a skin biopsy showed a nonspecific increase in melanin without other histopathological lesions. Because of the combination of hyponatremia, good glucose control, asthenia, low blood pressure and facial hyperpigmentation, a serum sample was obtained for cortisol measurement. This showed that the levels of this hormone were low, at 1.9 mcg/dl (normal level in the mornings: 5-25 mcg/dl), as were the levels of dehydroepiandrosterone, 1.7 ng/ml (normal: 2.5-6.5 ng/ml), and dehydroepiandrosterone-sulfate, 187 ng/ml (normal: 354-2560 ng/ml). A diagnosis of Addison’s disease was made. The eosinophil count was 600 cells/mm^3^. Computed tomography showed bilateral enlargement of the adrenal glands, with preserved outlines.

Glucocorticoid replacement was then started and the patient had a good outcome. She recovered from the asthenia and fatigue, her blood pressure and serum sodium returned to normal levels, and insulin administration was also resumed. Anti-tuberculosis drug treatment was resumed and, after two months, the regimen was reduced to isoniazid and rifampicin. This regimen was continued until completion of nine months of treatment.

At the end of the treatment, the ulcerated lesions had all healed and the nodules had resolved with only severe hyperpigmentation remaining. Three months following cessation of therapy, no new lesions had occurred and the patient remained well.

## DISCUSSION

In this paper, we reported the first case of a patient with EIB who received anti-tuberculosis drugs and developed adrenal insufficiency. A search for other case reports in the PubMed/Medline, Embase, Cochrane and Lilacs databases did not find any articles, as shown in Table 1.

**Table 1.** Total numbers of papers indexed in the Lilacs (Literatura Latino-Americana e do Caribe em Ciências da Saúde), Embase (Excerpta Medica), Medline and Cochrane Library databases in relation to the terms Bazin and adrenal insufficiency or Bazin and Addison’s disease

Data	Search term	Results
Medline (via Pubmed)	(Bazin OR (Erythema Induratum)) AND ((Adrenal Insufficiency) OR (Addisons Disease))	0 articles
Lilacs	(Bazin OR (Erythema Induratum) OR (Eritema Indurado) OR (Eritema Endurado)) AND ((Adrenal Insufficiency) OR (Enfermedad de Addison) OR (Doença de Addison) OR (Addisons Disease) OR (Insuficiencia Suprarrenal) OR (Insuficiencia Adrenal)	0 articles
Embase	(Bazin OR (Erythema Induratum)) AND ((Adrenal Insufficiency) OR (Addisons Disease))	0 articles
Cochrane Library	(Bazin OR (Erythema Induratum)) AND ((Adrenal Insufficiency) OR (Addisons Disease))	0 articles

Search conducted on September 17, 2011.

In 1861, Bazin gave the name erythema induratum to a nodular eruption that occurred on the lower legs of young women who had tuberculosis. He commented that the condition was not rare. Over the next 30 years, the disease attracted little notice, but with the discovery of the tubercle bacillus, interest revived and attempts were made, with varying success, to isolate the bacillus from the lesions.^1^

EIB is more frequently observed in females, in proportions of 9:1. The peak age of occurrence ranges from 20 to 30 years, and this period is characterized by painful nodules on the dorsal face of the legs, with diameters ranging from one centimeter to five centimeters. In 30% of the cases, these nodules may undergo necrosis and ulceration.^1^

The differential diagnoses include nodular vasculitis, papulonecrotic lichen, indurative tuberculosis of Darier-Roussy, necrobiosis lipoidica diabeticorum, erythema nodosum and Weber-Christian panniculitis.^1^ Our patient was diabetic, but the clinical presentation and biopsy findings from the lesion were not compatible with necrobiosis lipoidica diabeticorum.

After treatment with tuberculostatic drugs had been started, the patient developed clinical manifestations suggestive of adrenal insufficiency. This was confirmed as a diagnosis of Bazin disease, which could be made through the suggestive clinical and histopathological features, positive epidemiology for tuberculosis, positive tuberculin test and response to treatment with tuberculostatics.^2^ The response to anti-tuberculosis therapy confirmed the diagnosis, since untreated lesions persist for years.

An association between Bazin disease and foci of tuberculosis, such as in the lungs, pleura, pericardium, peritoneum, lymph nodes and endometrium, has been described in the medical literature. It has been estimated that, in 25 to 70% of the patients with EIB, Mycobacterium tuberculosis can be found in skin lesions through the polymerase chain reaction (PCR). In the remaining patients, the disease is considered to be idiopathic. In most cases, EIB occurs without demonstrable foci of tuberculosis infection, and the tuberculosis etiology is suggested by positive findings of purified protein derivative (PPD), as in the present case.^3^^,^^4^^,^^5^^,^^6^^,^^7^^,^^8^^,^^9^^,^^10^

After treatment with tuberculostatic drugs had been started, the patient developed clinical manifestations suggestive of adrenal insufficiency. This was confirmed by lower serum cortisol in the early morning. Because the patient’s cortisol level was lower than 3 mcg/dl, it was unnecessary to perform a cortrosyn stimulation test.^11^ It is worth noting that the adrenal failure developed after the anti-tuberculosis therapy had been started. Development of adrenal crises in patients who receive rifampicin has been described in the medical literature.^12^ Rifampicin increases hepatic steroid metabolism, which may lead to increased corticosteroid requirements in patients with adrenal insufficiency.^13^ This may have been the precipitating factor for the adrenal crisis in this particular case, given that is likely that our patient had inadequate adrenal reserves because of tuberculosis.

Tuberculosis is an important cause of adrenal insufficiency around the world, particularly in Brazil.^14^ We did not make measurements on antibodies to steroid 21-hydroxylase in order to rule out autoimmune adrenalitis, but the findings of bilaterally enlarged adrenal glands were suggestive of tuberculosis as the cause of adrenal failure.^15^^,^^16^

## CONCLUSIONS

The present study reported the clinical case of a patient with EIB who, after tuberculosis treatment, evolved with adrenal insufficiency. We emphasize that tuberculosis is still the main etiological factor for Addison’s disease, especially in underdeveloped countries.
